# Mechanical properties and microstructural analysis of MICP-reinforced coarse-grained saline soils under freeze-thaw cycling

**DOI:** 10.1371/journal.pone.0336266

**Published:** 2025-11-13

**Authors:** Liang Xiong, Lieyu Tian, Xiaolian Zhang, Mingxin Wang, Ailiyaer Ahemaiti

**Affiliations:** 1 Guangzhou Marine Geological Survey, China Geological Survey, Guangzhou, China; 2 Guangdong Bureau of Coal Geology, Guangzhou, China; Henan Polytechnic University, CHINA

## Abstract

Coarse-grained saline soils in cold regions, characterized by poor stability and low bearing capacity, pose serious risks to road and bridge infrastructure. Microbially Induced Calcium Carbonate Precipitation (MICP) is a sustainable geotechnical technique with potential for saline soil improvement, but its efficacy is compromised by high salinity and freeze-thaw cycling. In this study, sulfate coarse-grained saline soils with varying salt contents were reinforced via MICP and subjected to multiple freeze-thaw conditioning regimes. Mechanical properties and microstructural changes of the saline soils were characterized to elucidate the degradation of MICP-treated saline soils under coupled salinity and freeze-thaw effects. Results indicate that increasing salinity exerts both inhibitory and competitive effects on MICP, reducing calcium carbonate production by 80.97%. Increased numbers of freeze-thaw cycles exacerbated damage to the cementation network. Saline soils with Na_2_SO_4_ ≥ 6% exhibited markedly reduced resistance to degradation: porosity-reduction magnitude fell to 12.27% and cohesion decreased to 0.057 MPa. Furthermore, the coupled effects of salinity and freeze-thaw accelerated the loss of MICP’s ameliorative efficacy in saline soil.

## 1 Introduction

Under the combined influences of climate, hydrology, and anthropogenic activities [1,2], soils with a salt mass fraction exceeding 0.3% are classified as saline soils [3], which occur widely across the globe [4,5]. Coarse-grained saline soils exhibit pronounced collapse susceptibility and salt-induced swelling [6]; salt dissolution weakens interparticle cementation, while crystallization-driven expansion stresses disrupt soil structure [7]. Moreover, sulfates in coarse-grained saline soils absorb water and swell, and the degree of salt-induced swelling intensifies with increasing sulfate content [8,9]. Compared to other soluble salts in coarse-grained saline soils, sulfates more readily cause settlement, cracking, and collapse [10,11], posing serious risks to infrastructure, particularly road and bridge foundations [12,13]. Consequently, research into saline-soil amelioration techniques has become a focal topic.

Current research on saline soil amelioration has predominantly employed physical and chemical approaches [14,15]. Installation of drainage systems reduces salt migration and crystallization [16], while soil replacement directly eliminates salinization issues [17,18]. Soil amendments (such as biochar, humic acids, and bio-organic matter) lower soil salinity and improve nutrient status [19–21]; chemical stabilizers, including cement and coal fly ash, enhance soil structure and bearing capacity [22–24]. Amid growing emphasis on environmental sustainability and low-carbon development, Microbially Induced Calcium Carbonate Precipitation (MICP) has been introduced into saline soil treatment. MICP relies on microbial mineralization to form well-cemented calcium carbonate crystals and has been applied widely in geotechnical reinforcement, cultural-heritage consolidation, fracture sealing, soil-erosion mitigation and energy extraction [25–30]. Integrated MICP-based techniques that combine MICP with other materials or methods have also attracted considerable scholarly interest [31]. Two main strategies are used to apply MICP in saline soils: exogenous addition of cultured mineralizing microorganisms and nutrients [6,32,33], or in-situ stimulation of indigenous salt-tolerant mineralizers [34–36]. Hybrid approaches combining green materials with MICP are also under investigation [37]. However, most studies to date address low-salinity soils, and investigations of MICP in sulfate coarse-grained saline soil remain scarce.

Seasonally frozen regions also harbor widespread saline soils subjected to repetitive freeze-thaw cycles [38]. Researchers have found that freeze-thaw cycling disrupts the original fabric between soil particles, leading to the development of pores and cracks and causing soil property degradation [13,39,40]. In saline soils, this deterioration is exacerbated: warming intensifies moisture evaporation and drives continuous salt crystallization that severs interparticle bonds [41,42], while subsequent cooling induces salt recrystallization at undissolved crystals and generates volumetric expansion stresses from water phase change, further damaging soil structure and increasing porosity [3,43]. Consequently, seasonal freeze-thaw imposes a severe challenge to MICP-based stabilization of saline soils. Elucidating the mechanisms by which freeze-thaw cycling degrades MICP-reinforced saline soils is essential for assessing the long-term performance of MICP remediation.

This study applied MICP to reinforce sulfate coarse-grained saline soils with varying salt contents and subjected the treated samples to multiple freeze-thaw cycles based on the climatic conditions of the Turpan region in China. By characterizing changes in mechanical properties, pore structure, and microstructure before and after treatment, the degradation mechanisms induced by salinity and freeze-thaw cycles were investigated. The findings offer valuable guidance for the field implementation of MICP-based stabilization in cold, saline environments.

## 2 Materials and methods

### Materials

#### Cementation system.

*Sporosarcina pasteurii* (strain ATCC 11859), obtained from the China General Microbiological Culture Collection Center, was selected for reinforcing prepared coarse-grained saline soils due to its high salt and alkali tolerance [44]. *S. pasteurii* was inoculated into autoclaved liquid medium (Table 1) at a 1:100 (v/v) ratio. After incubation at 37°C with agitation for 24 h, the resulting bacterial suspension (OD₆₀₀ ≈ 1.1; urease activity ≈ 4.12 mM/min) was used for soil grouting. The cementation solution employed in all reinforcement tests comprised urea (0.5 M), calcium chloride (0.5 M), and deionized water.

**Table 1:** Composition of the liquid culture medium.

Casein peptone (g/L)	Peptone (g/L)	Urea (g/L)	NaCl (g/L)	Deionized water
5	15	20	5	—

#### Specimen preparation.

**Fig 1:**
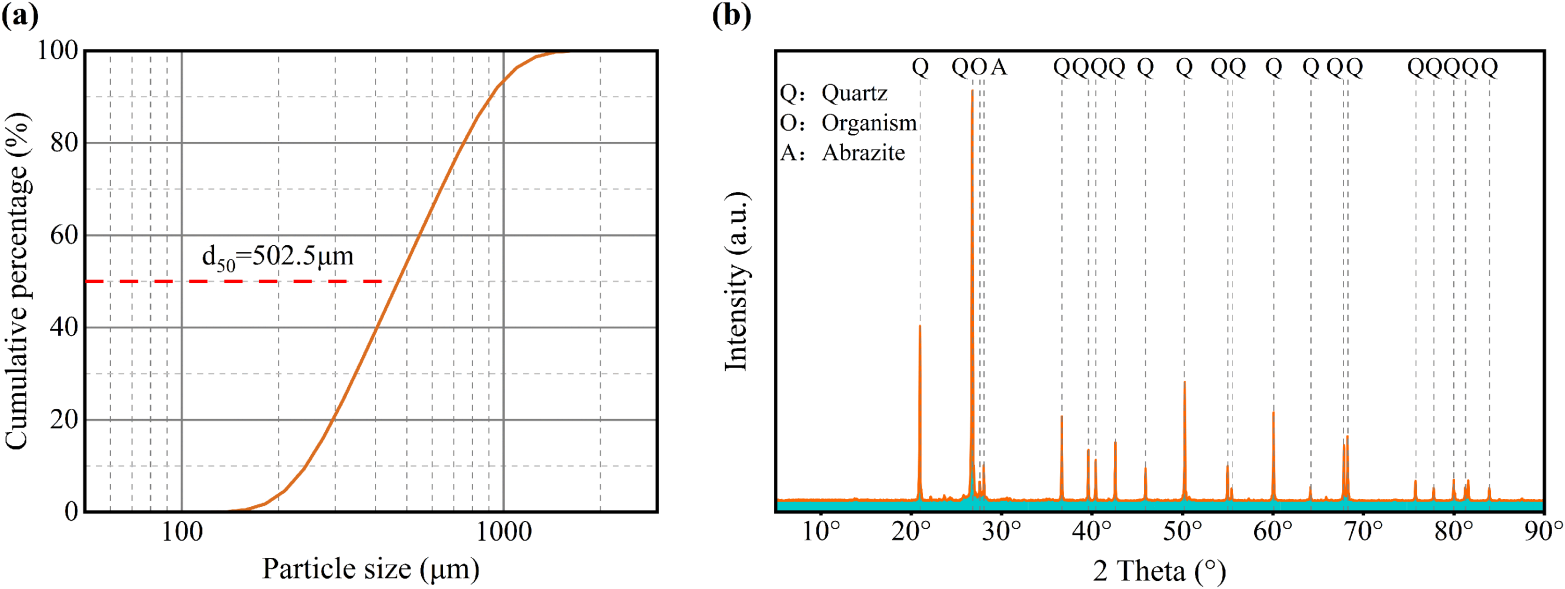
Particle size distribution curve (a) and XRD pattern (b) of standard siliceous sand. Particle size distribution of the standard siliceous sand was measured using a laser particle sizer. A 20-second laser scan of the sand sample produced particle size data, which were plotted as the particle size distribution curve **(a)**. X-ray diffraction data for the standard siliceous sand (b) were collected with an XRD diffractometer over a 2θ range of 5°–90° at a scan speed of 1°/min. The resulting diffractograms were processed using Jade software.

Sulfate-induced swelling in saline soils is generally more severe than that caused by other soluble salts [9]. Therefore, standard siliceous sand (GB/T 50123−2019) [45] (particle size distribution and composition shown in Fig 1; key parameters listed in Table 2) was mixed with Na_2_SO_4_ to prepare specimens. According to the Chinese Code for Investigation of Geotechnical Engineering [46], the prepared specimens are classified as coarse-grained saline soils. Wang et al. [47] reported that SO_4_^2-^ concentrations in typical saline soils of the Turpan region, China, are generally below 9%. Accordingly, four salinity gradients and four freeze-thaw conditioning regimes were established (Table 3). Specimen fabrication simulated the natural formation of saline soils: salt solutions of designated concentrations were poured into molds to fully submerge the sand samples, mechanically stirred, allowed to settle by natural sedimentation, and then subjected to isothermal dehydration and drying. Final specimen dimensions were 76 mm in height and 38 mm in diameter. In addition, blank control specimens without MICP treatment were prepared.

**Table 2:** The key properties and composition of standard siliceous sand.

Particle size (μm)	Dry density (g/cm^3^)	Quartz (wt%)	Organic matter (wt%)	Others (wt%)
150-1200	1.47-1.61	91.2	6.2	2.7

**Table 3:** Specimen preparation and freeze-thaw conditioning protocol.

Specimen number	Na_2_SO_4_ content (%)	Freeze-thaw cycle (times)	Specimen number	Na_2_SO_4_ content (%)	Freeze-thaw cycle (times)
S1	0	0	S9	6	0
S2	0	5	S10	6	5
S3	0	10	S11	6	10
S4	0	15	S12	6	15
S5	3	0	S13	9	0
S6	3	5	S14	9	5
S7	3	10	S15	9	10
S8	3	15	S16	9	15

### Experimental methods

#### Reinforcement test.

**Fig 2:**
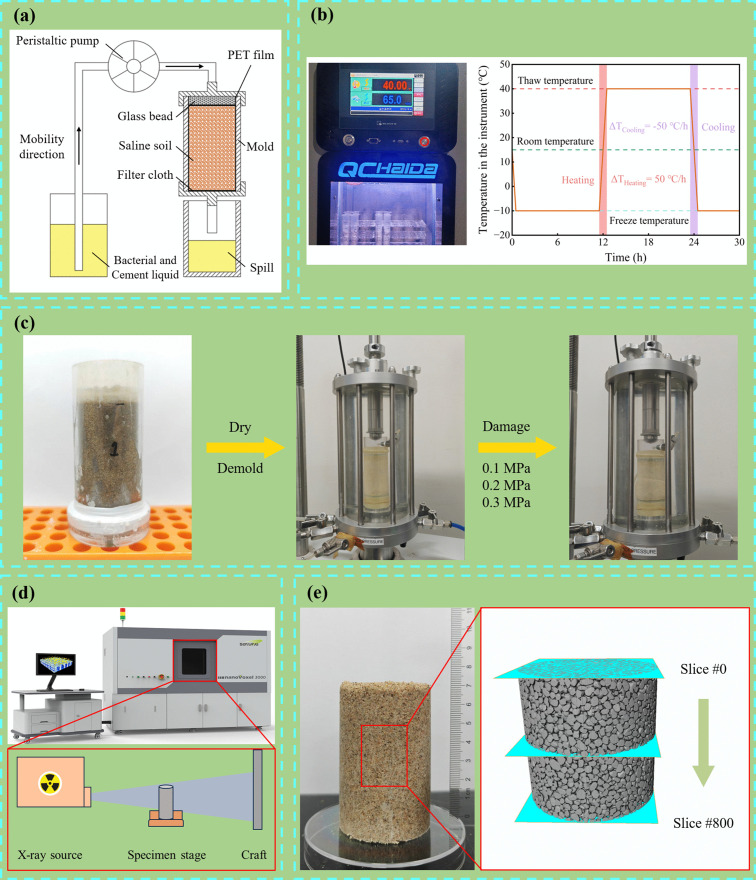
Primary Testing Methods: (a) MICP-based saline soil reinforcement apparatus; (b) Freeze-thaw conditioning chamber and single-cycle freeze-thaw protocol; (c) Consolidated undrained triaxial testing procedure; (d) Principles of X-ray computed tomography (XCT) testing; (e) Schematic of XCT result analysis.

An MICP-based saline soil reinforcement apparatus was designed (Fig 2a) by adapting the injection-grouting technique commonly used in civil engineering [48]. The system comprised a peristaltic pump (Renwei Fluid Co., Ltd., China), grout solutions, specimen molds, and overflow collection vessels. To improve grout distribution uniformity, glass microspheres and geotextile were placed inside the specimen mold, and a PET film liner was used to preserve specimen integrity during demolding. Cyclic grouting was used to reinforce the saline soil (grouting parameters in Table 4). A peristaltic pump was employed to introduce the bacterial suspension into the specimen from the top, followed by injection of the cementation solution. The volume of bacterial suspension and cementation solution was both approximately equal to the specimen’s pore volume. Under gravity, both fluids infiltrated the specimen pores and initiated biochemical reactions for soil consolidation. Upon completion of grouting, specimens were flushed with deionized water, dried, and demolded to obtain the reinforced specimens.

**Table 4:** Grouting parameters for reinforcement test.

Grouting rate (mL/min)	Grouting duration (min)	Grouting interval (min)	Interval between grouting cycles (h)	Number of grouting cycles (times)
4	9	30	12	10

#### Freeze-thaw cycle curing.

Saline soils are widely distributed in the Turpan region of China. Data from the China Meteorological Data Service Center (https://data.cma.cn/) indicate that winter and summer in the Turpan are relatively long and of approximately equal duration, while spring and autumn are comparatively short. In this region, the average monthly maximum temperature in summer reaches 40°C, while the average monthly minimum temperature in winter can drop to −10°C [49]. To simulate the winter low temperatures and summer high temperatures of the Turpan region, specimens of MICP-reinforced saline soil were subjected to freeze-thaw cycle curing using a freeze-thaw cycle chamber (Haida Instrument Co., Ltd., China). The procedure for a single freeze-thaw cycle is illustrated in Fig 2(b). The freezing temperature was set at −10°C and the thawing temperature at 40°C, with each temperature phase maintained for 11 hours. The heating and cooling phases were completed within 1 hour to simulate the rapid temperature fluctuations characteristic of spring and autumn. In addition, the relative humidity was set at 45% during the freezing phase and 65% during the thawing phase. This testing regime systematically simulates the effects of temperature variation, salt recrystallization, and humidity fluctuation on the mechanical properties and microstructure of MICP-reinforced saline soil.

#### Consolidated undrained triaxial tests.

Consolidated undrained (CU) triaxial tests were performed on the treated specimens using the fully automated triaxial system shown in Fig. 2(c) (Kangtuo Li Co., Ltd., Xi’an, China). Tests were conducted at confining pressures of 0.1, 0.2, and 0.3 MPa, with three replicate tests for each confining-pressure condition. Prior to shearing, each specimen was encased in a rubber membrane and consolidated under the target confining pressure. After consolidation, axial loading was applied at a strain rate of 1.0% of specimen height per minute (0.76 mm/min), in accordance with GB/T 50123−2019 [45]. The Stress and strain data for the specimen were recorded at 5-second intervals throughout the loading phase. Finally, the Mohr-Coulomb failure criterion was used to fit the peak stress values obtained at different confining pressures [50]. The corresponding strength envelope was plotted, and the mechanical parameters of the specimens were calculated.

#### X-ray computed tomography, XRD and SEM-EDS.

X-ray computed tomography (XCT) (Fig 2d) was employed to perform three-dimensional (3D) scanning on MICP-reinforced specimens after freeze-thaw cycle curing, scanning parameters are listed in Table 5. Subsequently, the acquired data volumes were reconstructed in Avizo software and partitioned into 801 slices from top to bottom using a thresholding approach (Fig 2e). This segmentation facilitated the quantitative calculation of changes in the specimen porosity, pore size distribution, and pore-throat connectivity. Furthermore, by utilizing the 3D pore network model, the influence mechanism of freeze-thaw action on the cementation matrix was analyzed. To evaluate permeability before and after treatment, the Absolute Permeability Tensor module in Avizo was used to simulate water flow through the pore network from the top to the bottom, thereby revealing the damage pathways of the cementation matrix induced by coupled salt-swelling and freeze-thaw effects.

**Table 5:** XCT scanning information and parameters.

Filter	Binning	Source voltage (kV)	Source current (mA)	Exposure (s)	Voxel size
The non-local means filter	1 × 1	150	60	0.6	Φ800 × 800

Samples were collected from the same depth within the reinforced specimens. X-ray diffraction (XRD) (Rigaku Corporation, Japan) was performed on these samples over a 2θ range of 5° to 90°, in order to identify the mineral composition and crystalline characteristics of the precipitates formed via MICP treatment. Concurrently, scanning electron microscopy (SEM) (ZEISS Instruments) was utilized to observe the cementation morphology between soil particles within the samples. This was coupled with energy-dispersive X-ray spectroscopy (EDS) (Oxford Instruments) to perform elemental mapping of selected areas, thereby determining the spatial distribution patterns of calcium carbonate within the microstructure.

## 3 Results

### Calcium carbonate content

**Fig 3:**
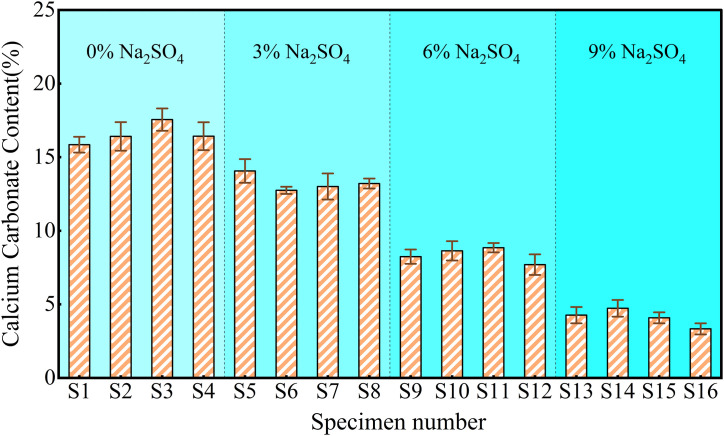
The calcium carbonate content of the specimens after MICP treatment. Samples were obtained from the upper, middle, and lower layers of the specimens. The mass loss percentage after reaction with HCl was calculated, and the average value obtained was considered as the calcium carbonate content of the specimen.

The calcium carbonate content of MICP-treated specimens was tested using the hydrochloric acid acidification method (Fig 3). As the salt content increased, the calcium carbonate content produced by *S. pasteurii* gradually decreased. The calcium carbonate content of unsalted specimens ranged from 15% to 18%, while that of specimens containing 9% Na_2_SO_4_ decreased to 3% to 6%. When Na_2_SO_4_ content exceeded 3%, the calcium carbonate content of the specimens significantly decreased (by approximately 50%), which can be attributed to the inhibition of microbial activity by high salt concentrations, thereby reducing the mineralization process.

### Mechanical properties of MICP-reinforced saline soil after freeze-thaw cycle curing

**Fig 4:**
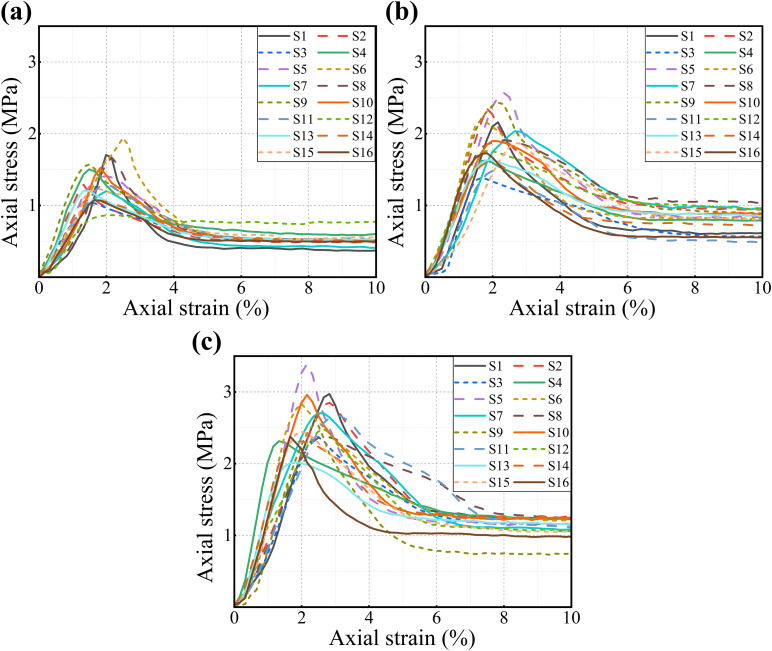
Axial stress-strain curves of treated specimens under different confining pressures: (a) 0.1 MPa; (b) 0.2 MPa; (c) 0.3 MPa.

The specimens untreated by MICP exhibited a loose state after demolding and were not subjected to consolidation undrained triaxial tests or XCT analysis. The stress-strain curves of the treated specimens under confining pressures of 0.1 MPa, 0.2 MPa, and 0.3 MPa are presented in Fig 4. The results indicate that both the salt content and the number of freeze-thaw cycles significantly affected the peak axial stress of the specimens. When the salt content was 0% or 3%, MICP technology effectively enhanced the mechanical properties of the treated specimens. Even after freeze-thaw cycle curing, these specimens maintained relatively high peak axial stress values. However, an increase in the number of freeze-thaw cycles still diminished the strength of the specimens. For example, the peak axial stress of specimen S3 decreased to 2.37 MPa, representing a reduction of 20.2%. Notably, in Fig 4(a), specimen S6 exhibits a higher peak stress than specimen S5, likely because initial ice formation within pore spaces temporarily increased specimen density. Furthermore, the strength of specimens with high salt content was significantly lower than those with low salt content and exhibited greater sensitivity to the number of freeze-thaw cycles. As observed in Fig 4(c), the peak stress of specimen S13 at a confining pressure of 0.3 MPa was only 2.02 MPa, which is 32% lower than specimen S1 under the same confining pressure. This demonstrates that increased salt content weakens the MICP reinforcement effect, consistent with the findings of Peng et al. [33]. Concurrently, freeze-thaw cycles further exacerbated the strength deterioration. After 15 freeze-thaw cycles, the peak stress of specimen S16 decreased to 2.38 MPa.

**Fig 5:**
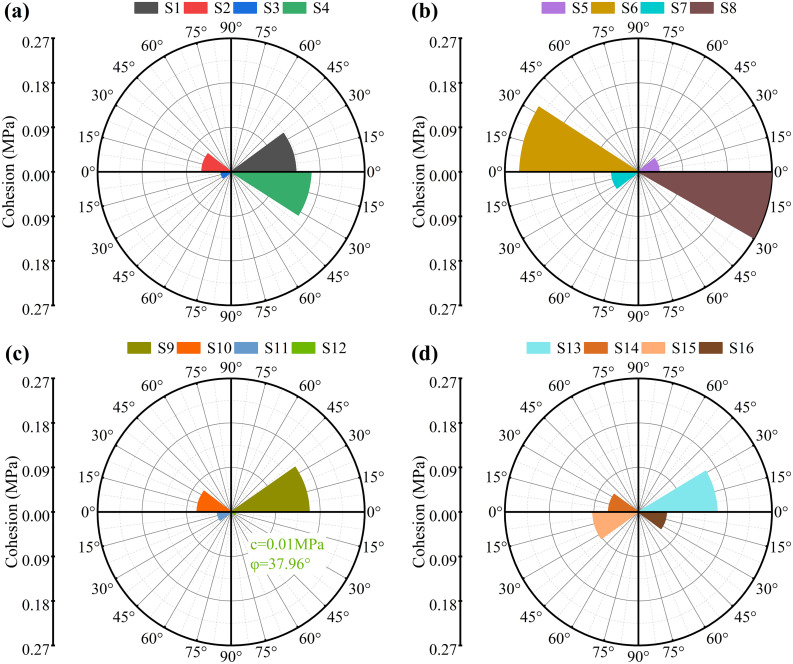
Cohesion and friction angle of treated specimens.

The cohesion and friction angle of each specimen, as shown in Fig 5, were derived from computational analyses. Specimen S1 exhibited a cohesion of 0.131 MPa and a friction angle of 36.24°, indicating that MICP markedly enhanced interparticle bonding and shear resistance. With increasing freeze-thaw cycles, cohesion declined sharply, whereas the friction angle initially increased before subsequently decreasing. At low numbers of freeze–thaw cycles, damage to the specimen’s cementation matrix remained limited. Pore filling by ice crystals likely imposed multiaxial stresses on the cementation matrix, increasing resistance to shear failure and thereby raising the friction angle. However, progressive fracture of the carbonate cement and the development of microcracks weakened cohesion, and particle rearrangement reduced shear resistance. High-salinity specimens displayed more pronounced degradation: for example, S13’s cohesion (0.159 MPa) and friction angle (31.20°) both fell below those of low-salinity specimens under the same conditions, likely due to reduced calcite precipitation and a weakened cementation network. Furthermore, extended freeze-thaw cycling exacerbated mechanical deterioration (S16’s cohesion dropped to 0.057 MPa) presumably because salt recrystallization and ice expansion jointly disrupted the cement matrix, causing interparticle bonds to fail.

### Pore structure evolution of MICP-reinforced saline soil after freeze-thaw cycle curing

**Fig 6:**
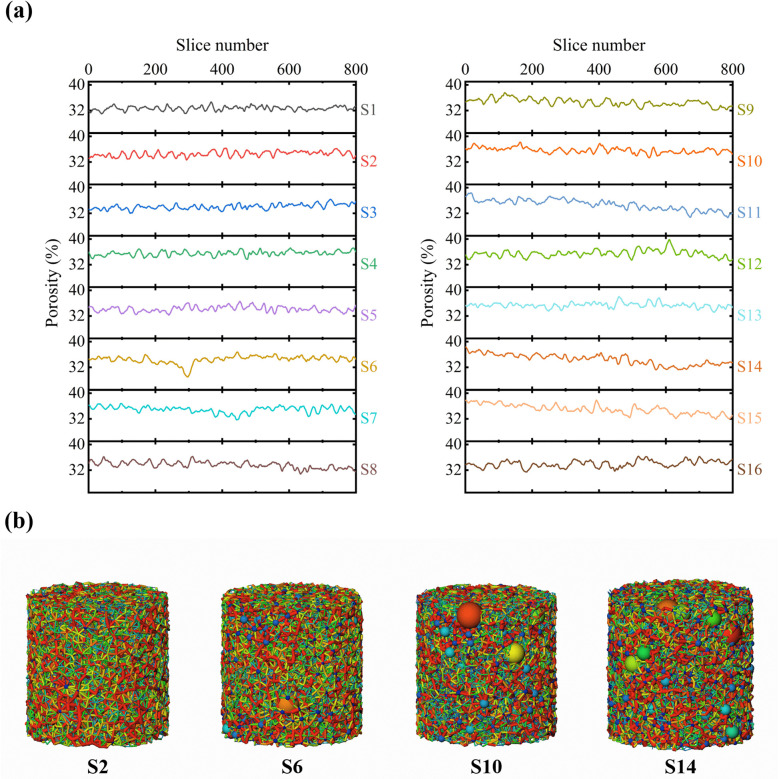
Layered porosity distribution (a) and pore structure model (b) of the untreated specimens.

The layered porosity distribution of specimens without MICP reinforcement or freeze-thaw conditioning is presented in Fig 6. Specimens S1-S4 exhibited relatively uniform porosity with no localized concentration zones, whereas saline specimens (e.g., S6) showed pronounced fluctuation, reaching a maximum difference of 7.77%. As salt concentration increased (specimens S9-S16), porosity variability intensified and displayed a clear top-to-bottom decline, indicating significant spatial heterogeneity with higher porosity near the specimen surface. This pattern is attributed to salt-induced swelling: during drying, moisture and dissolved salts migrate toward the surface, and crystallization generates expansion stresses that widen interparticle voids and increase pore volume. However, in high-salinity specimens S12-S14, porosity locally increased at the base, likely due to gravitational settling of salt crystals or localized dissolution-recrystallization effects [51].

**Fig 7:**
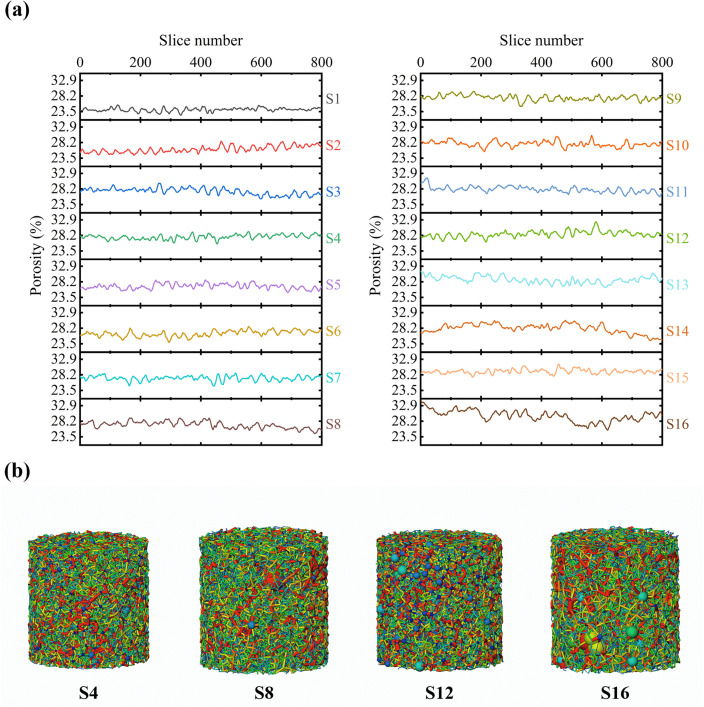
Layered porosity distribution (a) and pore structure model (b) of the treated specimens.

The layered porosity distribution of specimens following MICP treatment and freeze-thaw curing is shown in Fig 7. Biogenic calcium carbonate cementation led to a substantial reduction in porosity across all layers, demonstrating effective densification by MICP. In non-saline specimens, porosity declined uniformly and remained stable despite increasing numbers of freeze-thaw cycles. However, as salinity increased (specimens S5-S12), the overall reduction in porosity became progressively smaller; surface and base porosities were relatively constant, whereas mid-height porosity exhibited pronounced fluctuations with additional cycles. In the 9% Na_2_SO_4_ specimens, intense salt swelling produced highly irregular porosity profiles, and freeze-thaw cycling further amplified these variations: the surface porosity of specimen S16 rose from 33.67% to 34.30%, while base porosity also showed a slight increase. This pattern indicates that surface layers, subjected to larger thermal gradients and more frequent freeze-thaw events, experience more severe degradation of the cementation matrix, confirming the preferential deterioration of the specimen surface under coupled salt-swelling and freeze-thaw effects [52].

**Fig 8:**
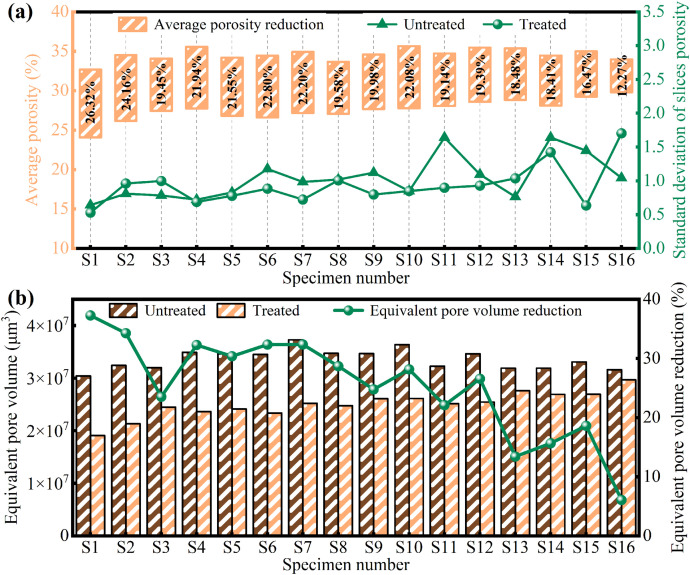
(a) Mean porosity and standard deviation of specimens before and after treatment; (b) Change in equivalent pore volume of samples before and after treatment.

The mean porosities and their standard deviations before and after treatment, calculated from stratified porosity profiles, are presented in Fig 8(a). Specimens with salt content below 6% exhibited a marked reduction in porosity and a more uniform pore distribution, whereas high-salinity specimens showed a substantially smaller porosity decrease and increased heterogeneity in pore structure. The cumulative effect of freeze-thaw cycling accentuated these trends, particularly in the high-salinity group. For example, specimen S14 experienced an 18.41% porosity reduction after five freeze-thaw cycles, and its standard deviation rose to 1.42, indicating pronounced pore structure heterogeneity. Notably, S16 displayed the highest standard deviation among all specimens, suggesting that the combined action of salt swelling and freeze-thaw cycling induced cement matrix detachment and the formation of large pores or cracks, thereby exacerbating structural instability.

Changes in equivalent pore volume of specimens before and after treatment are presented in Fig 8(b). Under identical freeze-thaw conditioning, the magnitude of pore volume reduction decreased as specimen salinity increased, with high-salinity specimens showing a steep drop in volume reduction at higher salt contents. At a given salinity level, additional freeze-thaw cycles induced significant pore regeneration due to ice-crystal expansion and salt migration fracturing the cementation matrix. For specimen S16, the equivalent pore volume declined from 3.16 × 10^7^ μm³ to 2.97 × 10^7^ μm³, representing only a 6.07% reduction. Moreover, after ten freeze-thaw cycles, specimens exhibited an initial rise followed by a decrease in pore-volume reduction, likely because the salt in S7 created an optimal osmotic pressure [53,54] that enhanced MICP consolidation and thereby mitigated pore structure degradation.

### Permeability changes in MICP-reinforced saline soil after freeze-thaw cycle curing

**Fig 9:**
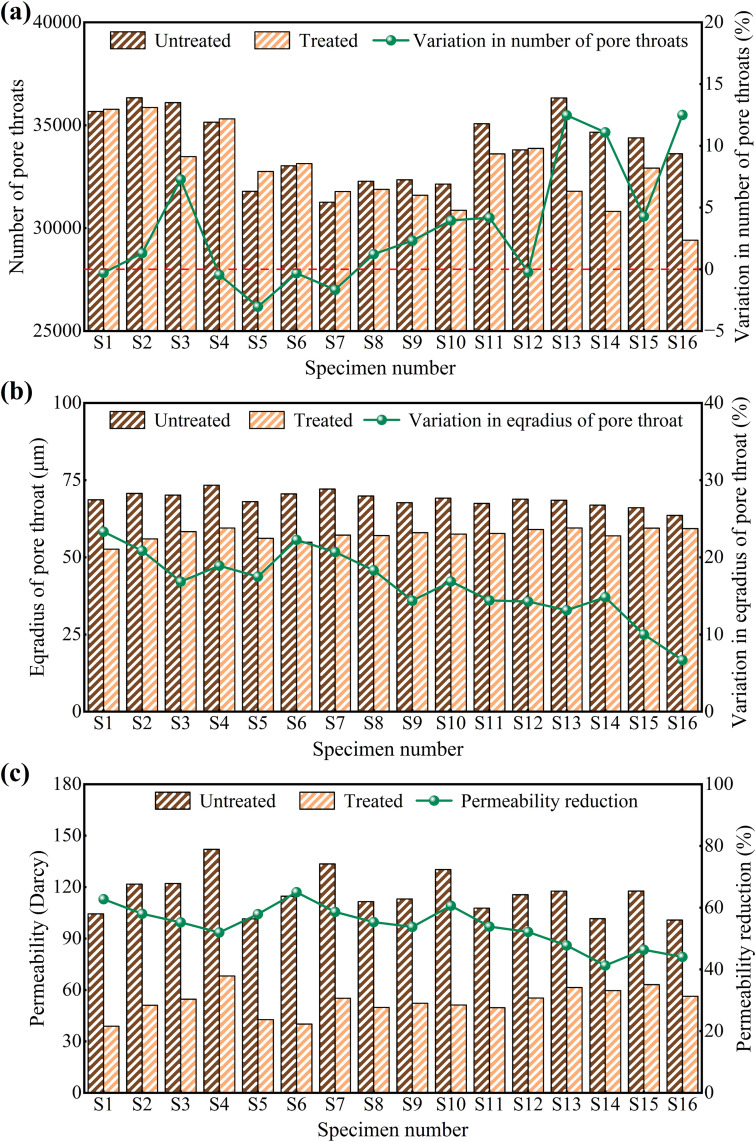
Number of throat openings (a), equivalent throat radius (b), and permeability (c) of specimens before and after treatment. Permeability values were converted using the factor 1 Darcy = 9.86923 × 10^−13^ m^2^.

Fig 9(a) presents the change in pore-throat count before and after treatment. In low-salinity specimens, the number of pore throats generally increased slightly after MICP treatment, and subsequent freeze-thaw cycles did not induce significant changes (Section 3.3). This behavior reflects the localized cementation by MICP-induced CaCO_3_, which subdivided existing pores into multiple smaller throats. In contrast, high-salinity specimens exhibited a pronounced decrease in pore-throat count: S9 showed a 2.32% reduction, while S13 declined by 12.48%. The greater loss of pore throats at higher salinities is attributed to competition between sulfate crystallization and biogenic cement for throat space, leading to complete blockage of some pore throats. Additionally, freeze-thaw cycling caused spalling of the cement matrix, further enlarging previously filled pores and reducing throat counts, which is similar with the finding of Liu et al. [55].

The changes in equivalent pore-throat radius before and after treatment are illustrated in Fig 9(b). In low-salinity specimens, the original pore throats exhibited contraction in radius due to filling by the cementation matrix. A reduction of 23.31% was observed for specimen S1. The reduction in pore-throat radius was less pronounced in high-salinity specimens. This is attributed to salt crystallization during the initial reinforcement stage hindering precipitate deposition on the pore-throat surfaces. However, salt recrystallization and ice crystal growth induced by freeze-thaw cycles further compromised the MICP reinforcement effect. Consequently, high-salinity specimens ultimately exhibited a diminished reduction in pore-throat radius. Furthermore, high salt content inhibited the growth of *S. pasteurii* and the mineralization process, reducing the effective precipitation of calcium carbonate within the pore throats. Under the synergistic effects of salt content and freeze-thaw action, the reduction in pore-throat radius decreased from 23.31% for specimen S1 to 6.69% for specimen S16.

Permeability before and after treatment (Fig 9c) was jointly controlled by pore volume, pore-throat count, and pore-throat radius. Low-salinity specimens exhibited a pronounced permeability decline: the permeability of S1 fell from 104.38 Darcy to 38.86 Darcy, a 62.77% reduction, owing to effective sealing of pore channels by calcium carbonate cementation, which impedes salt migration in saline soil [56]. High-salinity specimens also showed decreased permeability, but smaller reductions in pore volume and pore-throat radius limited the overall permeability drop.

### Characteristics of products in MICP-reinforced saline soil after freeze-thaw curing

**Fig 10:**
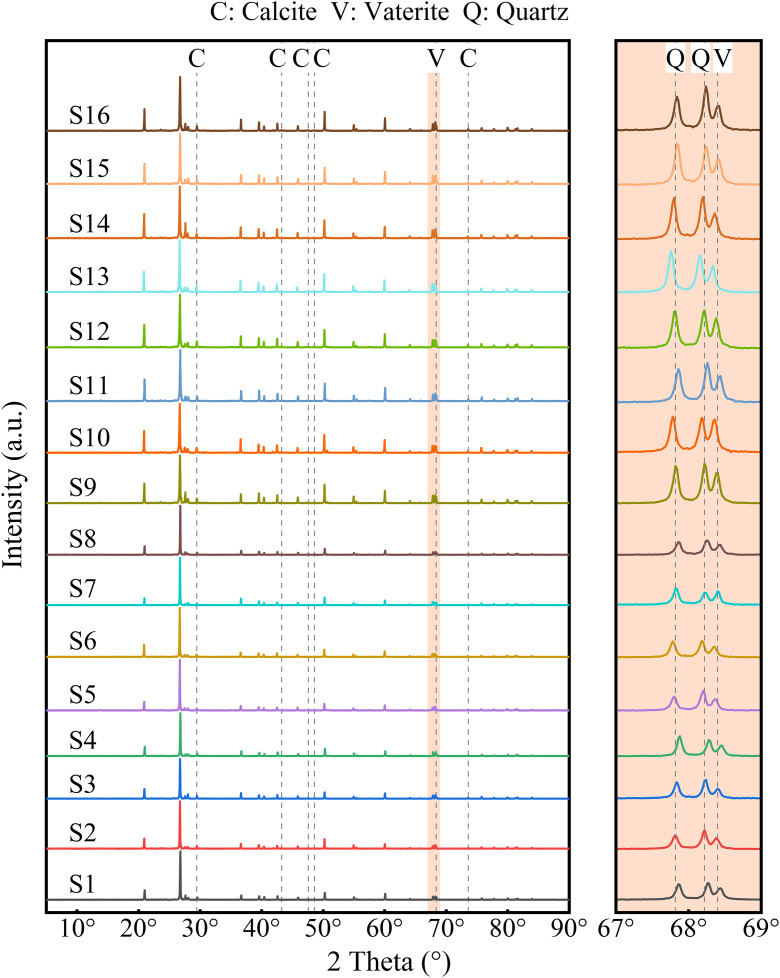
The XRD patterns of treated specimens.

Fig 10 presents the XRD patterns of MICP-reinforced saline soil specimens following freeze-thaw curing. All samples exhibited characteristic peaks of calcium carbonate. In comparison with the standard siliceous sand pattern (Fig 1b), both calcite and vaterite phases were identified; no aragonite peaks were detected. Calcite, as the thermodynamically stable phase, formed under the alkaline conditions generated by microbial metabolism [57]. Concurrently, localized supersaturation of pore solutions in saline soils created variable ion diffusion rates, leading to the formation of vaterite crystals [58].

**Fig 11:**
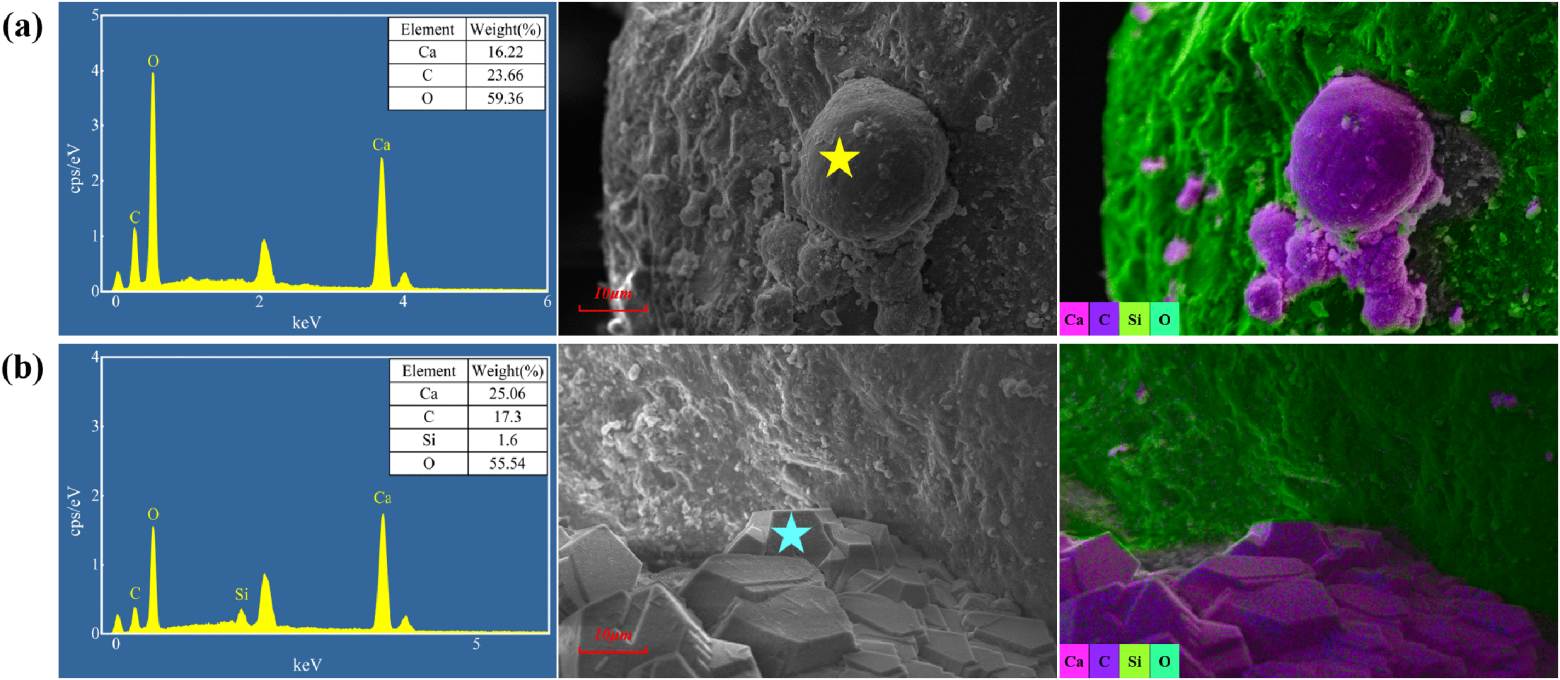
MICP-generated calcium carbonate: (a) vaterite; (b) calcite.

SEM-EDS confirmed that biogenic calcium carbonate occurred as vaterite spheres (Fig 11a) and calcite clusters (Fig 11b). Calcite predominantly localized at soil-particle contact points, whereas vaterite appeared as scattered deposits on particle surfaces. Noteworthy, no significant characteristic peaks for calcium sulfate were detected in any specimens. The sodium sulfate is thus inferred to have not participated substantially in the mineralization process, with its primary effects being the imposition of osmotic stress on *S. pasteurii* and the exacerbation of salt expansion in the saline soil specimens.

## 4 Discussion

### Deterioration mechanism of salt content on MICP-reinforced saline soil

**Fig 12:**
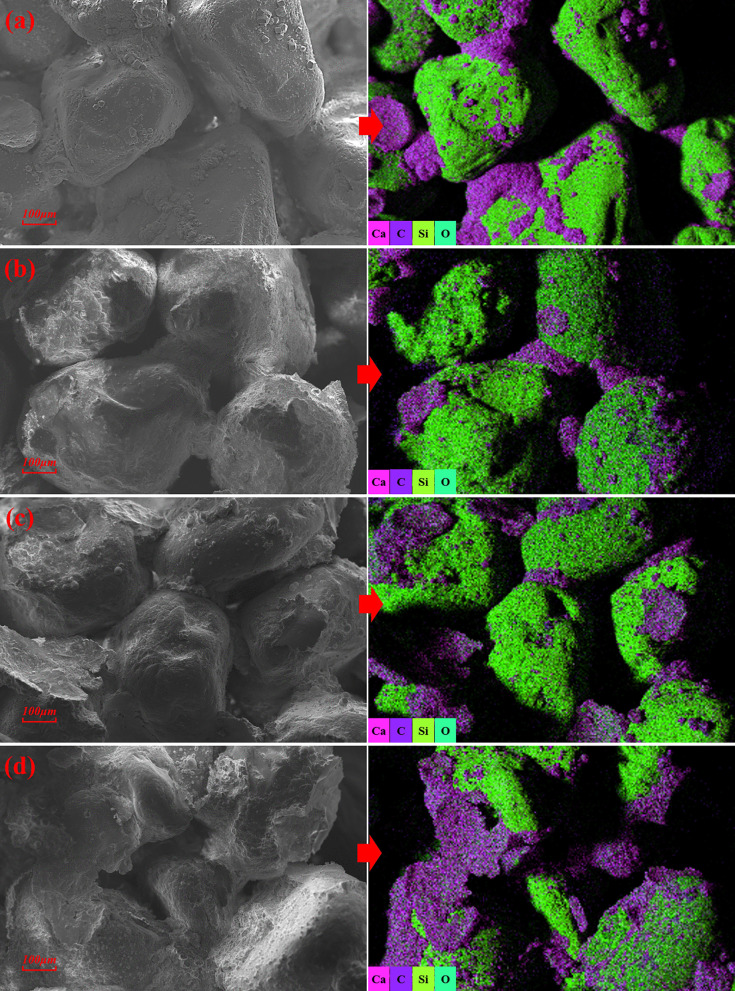
SEM-EDS results of different salt-containing specimens without freeze-thaw curing: (a) S1; (b) S5; (c) S9; (d) S13.

The SEM-EDS test results of the MICP-reinforced specimens without freeze-thaw treatment are shown in Fig 12. In specimen S1 (Fig 12a), MICP-generated CaCO3 formed calcite clusters at soil particle contacts and within pore spaces, while extensive areas of particle surfaces remained uncemented. In saline specimens S5 (Fig 12b) and S9 (Fig 12c), the overall coverage of calcium carbonate decreased and the density of cementation at particle contacts was reduced compared to S1, but the amount of vaterite on particle surfaces increased markedly. In S13 (Fig 12d), calcium carbonate predominantly appeared as surface coatings on soil particles, and cementation at contact points was substantially diminished, resulting in reduced reinforcement efficacy. Thus, in sulfate coarse-grained saline soil, increasing salt content shifts carbonate deposition from interparticle contacts and voids to particle surfaces, weakening bonding but strengthening coating effects and markedly increasing vaterite formation.

Considering porosity changes in specimens without freeze-thaw conditioning, salt content influences CaCO_3_ microstructure through competitive and inhibitory mechanisms. During soil drying, soluble salts preferentially crystallize at particle contacts and micropores, competing with MICP nucleation sites and redirecting biogenic CaCO_3_ cementation onto particle surfaces [59]. Concurrently, elevated salinity exerts significant inhibitory pressure on microbial metabolism, with higher salt concentrations causing stronger suppression [33]. Therefore, increasing salt content both reduces overall CaCO_3_ production by suppressing microbial growth and shifts cementation from interparticle bonding to surface bridging and coating. High-salinity conditions also amplify ion‐diffusion rate disparities [58], promoting greater vaterite formation. Mechanically, weakened bonding directly lowers cohesion, while enhanced surface coatings and increased vaterite roughness strengthen frictional resistance. This dynamic shift in the balance between cementation and friction underlies the cohesion and friction angle trends observed in Section 3.2.

### Deterioration mechanism of freeze-thaw cycles on MICP-reinforced saline soil

**Fig 13:**
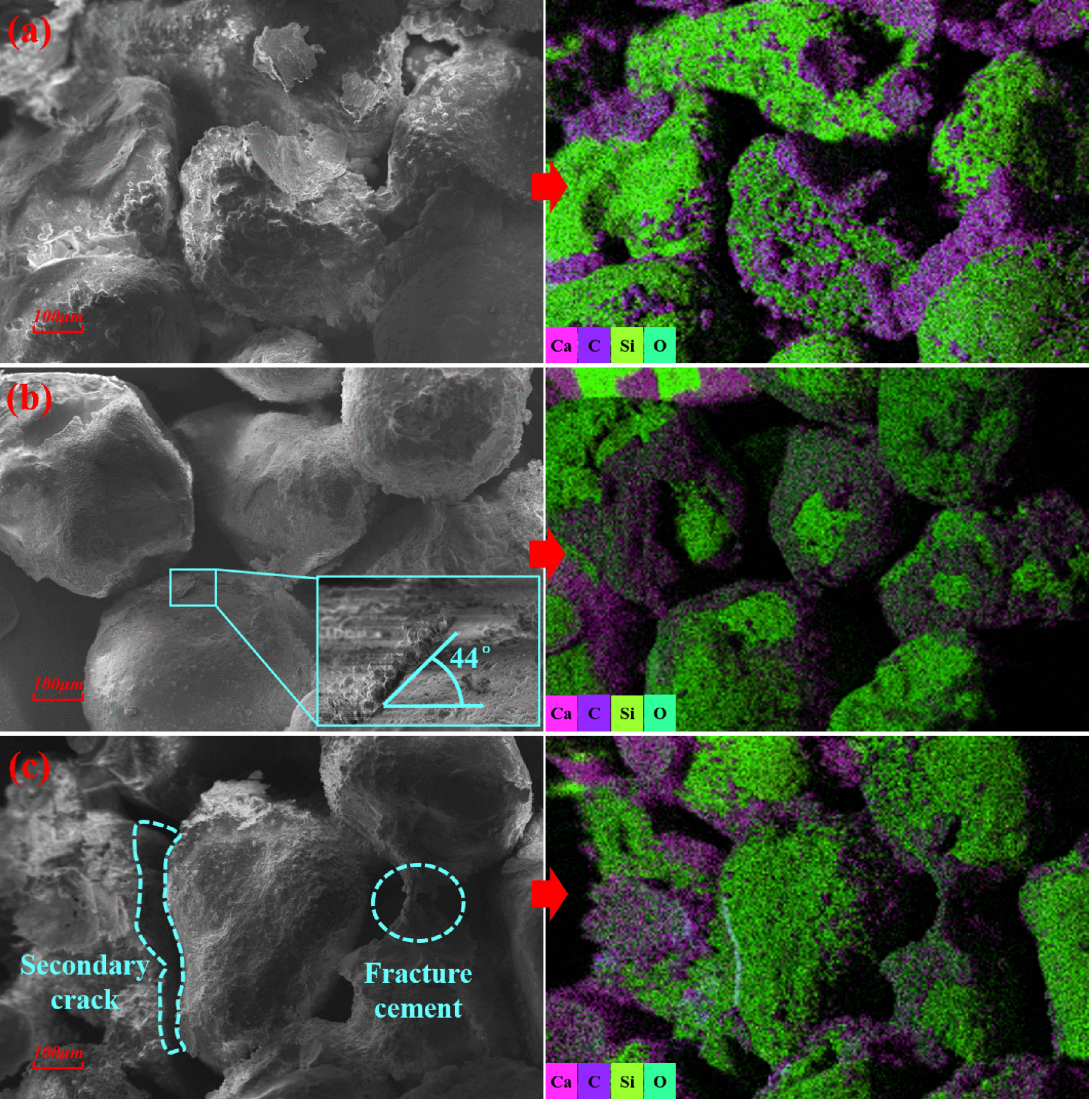
SEM-EDS results of specimens with 9% Na_2_SO_4_ after different freeze-thaw cycles: (a) S14; (b) S15; (c) S16.

Fig 13 shows SEM-EDS results for specimens with 9% Na_2_SO_4_ after varying numbers of freeze-thaw cycles. Although MICP-generated CaCO_3_ appeared within narrow pore throats, it predominantly coated soil particle surfaces that starkly contrasts with the distribution observed in low-salinity specimens. Comparing S13 (Fig 12d) and S14 (Fig 13a), both exhibited extensive CaCO3 coatings and vaterite on particle surfaces, with intact cementation in pore spaces. The calcium carbonate contents of S15 and S13 were both approximately 4%, yet the cohesion of S15 decreased by 42.02%. Fig 13(b) shows that CaCO3 precipitation within interparticle voids of S15 was markedly reduced, while CaCO3 coatings on particle surfaces became more uniformly distributed. Notably, localized detachment of CaCO3 films formed at shallow spallation angles without large-scale peeling or cracking. With further cycling (S16, Fig 13c), CaCO_3_ fragmentation intensified. Freeze-thaw conditioning caused partial dissociation of biogenic CaCO_3_, leading to secondary microcrack development and fracture of elongated cement bridges.

In non-saline specimens, MICP‐generated calcium carbonate preferentially occupies interparticle voids, reducing pore connectivity and preventing ice‐expansion stresses from exceeding the cementation network’s strength; consequently, these specimens exhibit no significant structural degradation during freeze-thaw cycles. In low-salinity saline soils, salt swelling is relatively minor, and ice-crystal expansion stresses during freeze-thaw cycles only partially weaken the strength of MICP cementation network, without inducing significant structural failure. By contrast, salt-induced swelling in saline soils intensifies with increasing salt concentration. Temperature and humidity fluctuations during freeze-thaw cycles further aggravate cycles of crystallization, dissolution. The recrystallization of water and dissolved salts, which can produce combined stresses from salt crystallization and ice expansion that compromise the integrity of the reinforced soil structure. Under salt-swelling and freeze-thaw coupling, CaCO_3_ in high-salinity specimens developed and propagated cracks, and even spalled, undermining reinforcement. As salinity and cycle count increase, degradation accelerates. Moreover, site competition by salts shifts the microbial cementation mode from bonding toward bridging, producing slender cement morphologies. Constrained by cross-sectional size effects [60], these elongated bridges exhibit reduced tensile strength against coupled stresses, ultimately leading to cement fragmentation.

## 5 Conclusions

This study focused on the evolution of pore structure and mechanical properties of MICP-reinforced sulfate coarse-grained saline soil under varying sulfate salt contents and numbers of freeze-thaw cycles. By integrating microstructural characterization, the deterioration mechanisms of MICP-reinforced coarse-grained saline soil subjected to freeze-thaw cycles were elucidated. The main conclusions are summarized as follows:

1. MICP effectively infilled the pore network of sulfate coarse-grained saline soils, thereby enhancing structural integrity and mechanical properties. After multiple freeze–thaw cycles, cohesion in high-salinity specimens remained as high as 0.092 MPa.
2. Salt content not only inhibits the growth of *S. pasteurii* but also competes with MICP for mineralization sites. This competition forces calcium carbonate crystals to precipitate more readily on particle surfaces rather than within constricted pore spaces. Consequently, the bonding effect of MICP is weakened, while its coating effect is enhanced.
3. Freeze-thaw cycling repeatedly generated ice-expansion stresses and amplified sulfate-induced swelling in coarse-grained saline soils, causing spalling, cracking, and fracture of MICP-produced calcium carbonate; such damage led to deterioration of pore structure and mechanical performance.
4. Saline soils with salt contents below 6% exhibited markedly greater resistance to freeze-thaw degradation following MICP treatment than those with salt contents ≥ 6%. Furthermore, both higher salt concentrations and an increased number of freeze-thaw cycles accelerated the deterioration of mechanical performance in MICP-reinforced coarse-grained saline soils.

The present findings provide a theoretical basis for applying MICP to stabilize saline soils in cold regions. In engineering applications, salt-tolerant microbial strains with enhanced temperature adaptability can be selected for mineralization, while simultaneously optimizing the reinforcement process to improve the controllability of reinforcement outcomes. Future work should develop a freeze-thaw damage model for MICP-treated saline soils that incorporates salt concentration, number of cycles, strength metrics, and pore-structure parameters to enable quantitative analysis of interactions among these factors. Additionally, expand the range of salinity gradients and increase the number of freeze-thaw cycles. Conduct comparative studies of MICP and alternative remediation methods. Such efforts will facilitate the translation of MICP into practical engineering solutions for saline-soil stabilization in cold environments.

## Supporting information

S1 Table Peak stress and standard deviation of specimens under different confining pressures. (DOCX)
