# Supplementary material for: Mechanical properties and microstructural analysis of MICP-reinforced coarse-grained saline soils under freeze-thaw cycling
Source: PLoS One. 2025 Nov 13;20(11):e0336266. doi: 10.1371/journal.pone.0336266 (PMC12614544; doi:10.1371/journal.pone.0336266)
Supplement: S1 Table — (DOCX) [file pone.0336266.s001.docx]

**S1 Table.** **Peak stress and standard deviation of specimens under different confining pressures.**

| **Specimen number** | **Confining pressure (MPa)** | **Group1** | **Group2** | **Group3** | **Mean (MPa)** | **Standard deviation (MPa)** |
| --- | --- | --- | --- | --- | --- | --- |
| S1 | 0.1 | 1.69 | 1.72 | 1.70 | 1.70 | 0.0134 |
| S2 | 0.1 | 1.32 | 1.33 | 1.21 | 1.29 | 0.0525 |
| S3 | 0.1 | 1.01 | 1.04 | 1.04 | 1.03 | 0.0147 |
| S4 | 0.1 | 1.47 | 1.51 | 1.53 | 1.50 | 0.0257 |
| S5 | 0.1 | 1.37 | 1.42 | 1.44 | 1.41 | 0.0309 |
| S6 | 0.1 | 1.99 | 1.95 | 1.88 | 1.94 | 0.0446 |
| S7 | 0.1 | 1.15 | 1.22 | 1.22 | 1.20 | 0.0323 |
| S8 | 0.1 | 1.62 | 1.75 | 1.63 | 1.67 | 0.0571 |
| S9 | 0.1 | 1.53 | 1.65 | 1.54 | 1.57 | 0.0565 |
| S10 | 0.1 | 1.56 | 1.49 | 1.52 | 1.52 | 0.028 |
| S11 | 0.1 | 1.32 | 1.27 | 1.28 | 1.29 | 0.0223 |
| S12 | 0.1 | 0.91 | 0.88 | 0.80 | 0.87 | 0.0481 |
| S13 | 0.1 | 1.24 | 1.23 | 1.15 | 1.21 | 0.0411 |
| S14 | 0.1 | 1.00 | 1.05 | 1.21 | 1.09 | 0.0877 |
| S15 | 0.1 | 1.18 | 1.23 | 1.28 | 1.23 | 0.0384 |
| S16 | 0.1 | 1.11 | 0.99 | 1.13 | 1.08 | 0.0606 |
| S1 | 0.2 | 2.28 | 2.14 | 2.06 | 2.16 | 0.0907 |
| S2 | 0.2 | 2.42 | 2.34 | 2.30 | 2.35 | 0.0495 |
| S3 | 0.2 | 1.37 | 1.30 | 1.48 | 1.38 | 0.0738 |
| S4 | 0.2 | 1.66 | 1.54 | 1.68 | 1.63 | 0.0614 |
| S5 | 0.2 | 2.52 | 2.49 | 2.71 | 2.57 | 0.0955 |
| S6 | 0.2 | 2.15 | 2.10 | 2.32 | 2.19 | 0.0955 |
| S7 | 0.2 | 1.96 | 2.01 | 2.14 | 2.04 | 0.0755 |
| S8 | 0.2 | 1.87 | 2.01 | 1.85 | 1.91 | 0.0717 |
| S9 | 0.2 | 2.42 | 2.49 | 2.39 | 2.43 | 0.0405 |
| S10 | 0.2 | 1.98 | 1.84 | 1.88 | 1.90 | 0.059 |
| S11 | 0.2 | 1.55 | 1.44 | 1.50 | 1.50 | 0.045 |
| S12 | 0.2 | 1.74 | 1.82 | 1.72 | 1.76 | 0.0423 |
| S13 | 0.2 | 1.67 | 1.69 | 1.54 | 1.63 | 0.0647 |
| S14 | 0.2 | 1.66 | 1.56 | 1.58 | 1.60 | 0.0426 |
| S15 | 0.2 | 1.81 | 1.91 | 1.88 | 1.87 | 0.0421 |
| S16 | 0.2 | 1.65 | 1.71 | 1.83 | 1.73 | 0.0761 |
| S1 | 0.3 | 3.10 | 2.87 | 2.95 | 2.97 | 0.0954 |
| S2 | 0.3 | 2.91 | 2.81 | 2.83 | 2.85 | 0.0433 |
| S3 | 0.3 | 2.33 | 2.28 | 2.51 | 2.37 | 0.0978 |
| S4 | 0.3 | 2.26 | 2.30 | 2.38 | 2.31 | 0.0499 |
| S5 | 0.3 | 3.41 | 3.29 | 3.47 | 3.39 | 0.0752 |
| S6 | 0.3 | 2.90 | 2.77 | 2.85 | 2.84 | 0.0536 |
| S7 | 0.3 | 2.66 | 2.75 | 2.71 | 2.71 | 0.0369 |
| S8 | 0.3 | 2.44 | 2.39 | 2.31 | 2.38 | 0.0519 |
| S9 | 0.3 | 2.62 | 2.48 | 2.50 | 2.53 | 0.0611 |
| S10 | 0.3 | 3.10 | 2.87 | 2.91 | 2.96 | 0.1009 |
| S11 | 0.3 | 2.73 | 2.76 | 2.51 | 2.67 | 0.1102 |
| S12 | 0.3 | 2.62 | 2.43 | 2.39 | 2.48 | 0.1005 |
| S13 | 0.3 | 1.89 | 1.96 | 2.21 | 2.02 | 0.138 |
| S14 | 0.3 | 2.41 | 2.27 | 2.25 | 2.31 | 0.0721 |
| S15 | 0.3 | 2.36 | 2.46 | 2.44 | 2.42 | 0.044 |
| S16 | 0.3 | 2.43 | 2.31 | 2.40 | 2.38 | 0.0508 |
